# The Impact of Actigaming on Emotional Attentional Biases in College Students: An Exploratory Crossover Trial

**DOI:** 10.3390/brainsci16020170

**Published:** 2026-01-30

**Authors:** Xiaofen Ding, Jinlong Wu

**Affiliations:** 1 Hunan First Normal University, Changsha 410205, China; 2 College of Physical Education, Southwest University, Chongqing 400715, China

**Keywords:** actigaming, aerobic bicycle, emotional attentional biases, mood state, college student

## Abstract

**Background:** Prior research has provided evidence supporting the claim that actigaming may offer benefits for individuals’ emotions. However, the impact of actigaming on emotional attentional biases remains uncertain. **Objective:** To address this gap in the literature, this study aims to examine whether actigaming affects emotional attentional biases. **Methods:** We conducted a randomized crossover experiment in this study. A total of 18 college students completed a single session of actigaming and aerobic bicycle exercise for 40 min. Before and after the exercises, each participant completed the Profile of Mood States Questionnaire (POMS), Emotion Regulation Questionnaire (ERQ), and emotional attentional biases task. The heart rates of study participants were measured during the intervention to determine their maximum and average rates. **Results:** The following two findings were identified in this study: (1) There are no significant differences in maximum heart rate and average heart rate between participants of the aerobic bicycle intervention and the actigaming intervention (*p* = 0.352; *p* = 0.643, respectively). (2) Cores of POMS under the baseline condition present no significant difference between the two interventions (*p* = 0.872). However, the POMS post-test scores of participants in the actigaming intervention are significantly lower than those of participants in the aerobic bicycle intervention (*p* = 0.002). The main finding of the study is that, under baseline conditions, there are no significant differences in emotional attentional bias scores between the two interventions (*p* = 0.352). However, post-test scores show that participants in the actigaming intervention exhibit significantly lower attentional bias toward negative emotions compared to those in the aerobic bicycle intervention (*p* = 0.001). **Conclusions:** Actigaming more favorably post-exercise mood and significantly attenuates attentional bias toward negative stimuli compared with aerobic cycling. Therefore, the results of this study need to be confirmed by higher-quality studies in the future.

## 1. Introduction

An individual’s tendency to prioritize emotionally salient information within a complex sensory environment is referred to as emotional attentional bias [1]. Such prioritization confers advantages in attentional allocation and information processing [2]. And supports the rapid and efficient appraisal of emotional events [3]. Crucially, a sustained bias toward negative information is not merely an epiphenomenon of psychopathology; rather, it is a core cognitive factor implicated in the onset, maintenance, and recurrence of several disorders [4,5], with robust links to anxiety and depression [6,7]. Accordingly, delineating the mechanisms that shape emotional attentional biases has clear theoretical and translational significance for mental health and everyday functioning [8,9].

Converging evidence suggests that physical activity—particularly aerobic exercise—can modulate these biases. For example, following moderate-intensity exercise, participants exhibit greater attentional bias toward pleasant relative to unpleasant faces, and the bias toward unpleasant faces is attenuated during exercise compared with rest [10]. In clinical samples, exercise has been shown to facilitate disengagement from threat and reorient attention more effectively than control conditions [11]. Taken together, these findings indicate that physical activity can rebalance attentional allocation away from negative and toward positive emotional stimuli, highlighting exercise as a viable behavioral regulator of emotion-cognition interactions.

Against this backdrop, actigaming has emerged as a technology-enhanced exercise modality that integrates physical and/or motor-cognitive activity with interactive game mechanics to increase energy expenditure and cognitive engagement. Typical implementations include dance, skiing, boxing, and bowling exergames that emphasize movement–game integration to enhance enjoyment and motivation. Within this umbrella, actigaming denotes structured, goal-directed interventions aimed at improving task-specific performance (e.g., sport skills) or domain-specific outcomes (e.g., muscular power, selective attention, working memory) [12,13,14]. Relative to conventional exercise or therapy, actigaming consistently yields higher enjoyment, which is associated with better adherence and potentially greater effectiveness over time [15,16].

Comparative work further suggests emotional benefits specific to actigaming. Combining virtual reality with cycling increases exercise enjoyment and interest beyond traditional approaches [17,18], and dance-based actigaming has been associated with lower fatigue than traditional aerobic dance [19]. A recent systematic review synthesizing multiple trials reports that actigaming reduces anxiety, depression, and stress while enhancing relaxation, pleasure, mood, vigor, and subjective well-being; moreover, gamified feedback appears to bolster self-efficacy, self-esteem, and perceived behavioral control, thereby augmenting perceived energy and resilience [20]. Collectively, these data position actigaming as a promising platform for emotion promotion in both leisure and clinical contexts.

However, despite robust evidence of affective improvements, it remains unclear whether actigaming specifically alters attentional allocation to emotional stimuli. Most prior studies have focused on mood or general affect rather than on bias-sensitive attentional metrics, and direct head-to-head comparisons with conventional aerobic exercise are scarce. To address this gap, the present study will compare the effects of actigaming and traditional aerobic exercise on shifts in attention toward emotional information, using behavioral indices of emotional attentional bias.

## 2. Methods

### 2.1. Study Participants

**Figure 1:**
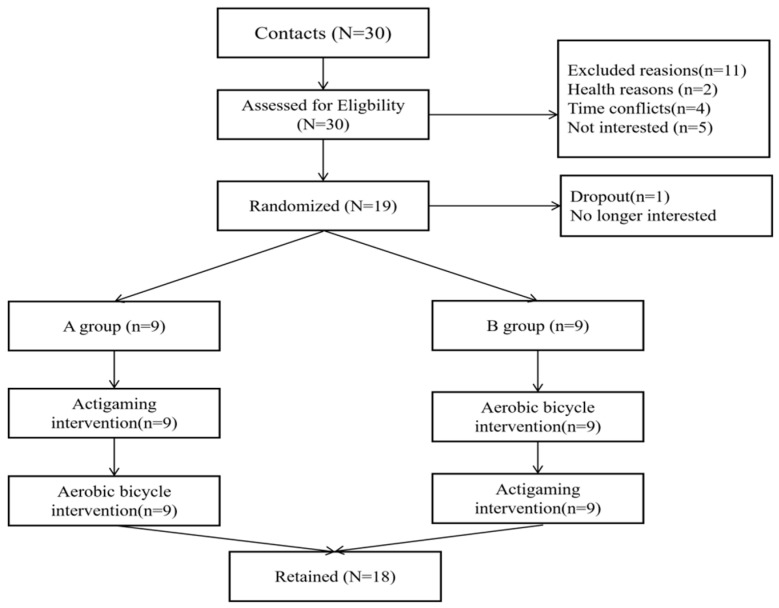
Detailed flow of participants through the study following CONSORT guidance.

This study was a randomized crossover study with an unblinded intervention and a blinded outcome assessment of the effects of a single session of actigaming on emotional attentional biases in college students. This study utilized a convenience sample of 18 college students, who were recruited from Southwest University through online advertisements and posters, with their detailed demographic characteristics shown in Table 1. The inclusion criteria for the study participants are as follows: (1) full-time university students aged 18–35; (2) no self-reported diagnosed physical and/or mental disabilities; (3) no contraindication for exercise; (4) no regular exercise performed within the past week. The exclusion criteria are as follows: (1) individuals who have taken psychiatric medication within the past 3 months; (2) individuals with self-reported, diagnosed physical and/or mental disabilities; (3) individuals with any contraindication to exercise; (4) Individuals who engaged in regular exercise within the past week (Figure 1 showed the detailed flow of participants).

To control external factors that could potentially impact their mood state, all participants were instructed to refrain from engaging in physical exercise, consuming alcohol, and consuming caffeine for a minimum of twenty-four hours before the commencement of the experiment. This study was approved by the Ethics Committee of Southwest University School of Physical Education (No. SWU-20230508-C1). The researchers obtained informed consent from all participants to take part in the study and ensured data privacy and confidentiality.

### 2.2. Experimental Procedure

**Figure 2:**
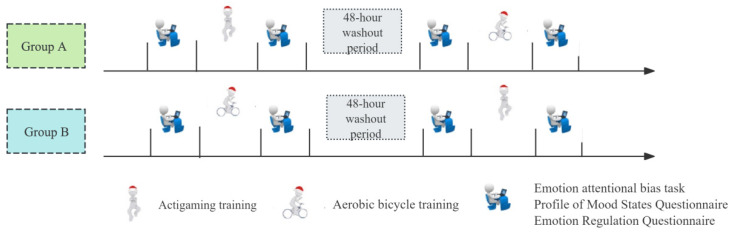
The flowchart of the randomized crossover experiment in this study.

Approximately three days (±1 day) before the commencement of the experiment, the 18 participants were introduced to the experimental procedures (Figure 2). The researcher provided a detailed explanation of the experimental process and arranged two separate groups (Group A and Group B). Using the random number generator (sample() function) in R software, version 4.1.0, participants were randomly assigned to one of the two experimental groups. Allocation sequences were placed in opaque, sealed envelopes and only opened after each participant completed baseline assessments and provided informed consent, thereby maintaining allocation concealment. In this crossover design, Group A (n = 9) received the actigaming intervention in Period 1 and the aerobic bicycle intervention in Period 2, while Group B (n = 9) received the interventions in the opposite order. A 48 h washout period was imposed between sessions to minimize carryover effects [21,22].

The intervention time was fixed from 2:00 p.m. to 5:00 p.m. Participants rested briefly upon entering the lab to avoid mood or heart rate fluctuations during travel. After resting, heart rate was recorded by using a sport-tester (Polar Electro, Helsinki, Finland), which was used to record resting heart rates of participants, who then completed the pretest sections of the questionnaire and cognitive task. Each participant performed a 5 min warm-up exercise in the laboratory, consisting of stretching of the upper and lower limb muscles, followed by a 30 min actigaming or aerobic bicycle exercise, and a 5 min relaxation exercise consisting of stretching of the upper and lower limb muscles. After the relaxation exercise, participants were allowed to rest for 5 min on the chair [23]. When the resting heart rates of participants returned to their normal levels (or within a range of ±10%), they were required to complete the post-test sections of the questionnaire (1. POMS; 2. ERQ) and the cognitive task (emotion attentional bias task). Both experiments were conducted at the sports rehabilitation laboratory of Southwest University.

### 2.3. Measurement Tool

(1) Profile of Mood States Questionnaire

The Chinese Version of the Profile of Mood States Questionnaire (POMS) [18] was used to assess the mood states of participants. The scoring criterion for this questionnaire is as follows: “Almost not” = 0; “A little bit” = 1; “Moderate” = 2; “Quite a lot” = 3; and “Extremely strong” = 4. Raw scores for each of the seven subscales were summed, and their T-scores were calculated through reference norms. A total mood score was calculated by subtracting the sum of the scores for two positive moods (vigor and self-esteem) from the sum of the scores for five negative moods, with the addition of 100. A high total mood score represents a negative mood state of the participant including tension, anger, fatigue, depression, and confusion), while a low total mood score represents a better positive mood state of the participant including vigor. The Chinese Version of the POMS measure showed high reliability (Cronbach α = 0.83) in Chinese participants [24].

(2) Emotion Regulation Questionnaire

The Chinese Version of the Profile of Emotion Regulation Questionnaire (ERQ) [25] was used in this study to assess the emotion regulation strategies of participants. This scale uses six items to evaluate cognitive reappraisal and four items to evaluate expressive suppression. Each item follows a scoring criterion: “strongly disagree” is scored with 1, “Strongly agree” is scored with 7, and so on. Also, a higher score indicates more frequent use of the emotional regulation strategy by the participant. The Chinese Version of ERQ is a reliable and effective self-report measure of emotion regulation strategies in Chinese participants [26].

(3) Emotion Attentional Bias Task

Pictures were selected from the Chinese Facial Affective Picture System (CFAPS) [27] as picture stimuli used in this study. With a score scale of 1–9, the valences, pleasure, and arousal of these pictures were scored by thirty-seven college students aged 18 to 35 years with no mental disorder. Face pictures with 85% valence consistency and pleasure within a range of M ± SD were selected as pictures to be used in this experiment, with their arousal matched with each other. Finally, twenty-five disgusted and twenty-five pleasant face pictures, along with one hundred neutral face pictures, were selected, resulting in two interventions of one hundred disgusted-neutral face pairs and one hundred pleasant-neutral face pairs. Disgusted-neutral face pairs presented significant differences in their pleasantness (M_disgust_ = 2.99 ± 0.56, M_neutral_ = 4.25 ± 0.54, t = 10.34, *p* = 0.002) and arousal (M_disgust_ = 6.31 ± 0.94, M_neutral_ = 3.76 ± 0.60, t −16.7035, *p* = 0.046). Pleasant-neutral face pairs also have significant differences in their pleasantness (M_pleasure_ = 5.93 ± 0.90, M_neutral_ = 4.25 ± 0.54, t = −11.99, *p* = 0.027) and arousal (M_pleasure_ = 5.01 ± 1.13, M_neutral_ = 3.76 ± 0.60, t = −7.62, *p* = 0.034). The experimental program was designed with E-Prime 1.1 software, and picture stimuli were displayed on a 15-inch computer screen. Participants sat 60 cm away from the computer screen, with their eyes parallel to the center of the screen, and horizontal and vertical visual angles of each picture at around 6.4 and 8.2, respectively.

A modified dot-probe task method was applied in this study. A black “+” fixation point first appeared in the center of the white computer screen. After 500 ms, two face pictures with a size of 10.9 cm × 9.1 cm and a distance of 2.5 cm were presented on the left and right sides of the fixation point, with equal distances from the fixation point. These two pictures disappeared after 500 ms. Then, dots “·” were presented on the left and right sides of the fixation point (where face pictures were previously shown) as detection stimuli. Participants were required to judge the positions of the dots, with their left index fingers pressing the “F” key when the dot appeared on the left side of the fixation point and their right index fingers pressing the “J” key when the dot appeared on the right side of the fixation point. After that, the detection stimuli and the fixation point disappeared, followed by a 500 ms period of blank screen before the next trial. There were a total of two hundred trials in the task, with a rest period between every one hundred trials [27,28].

**Figure 3:**
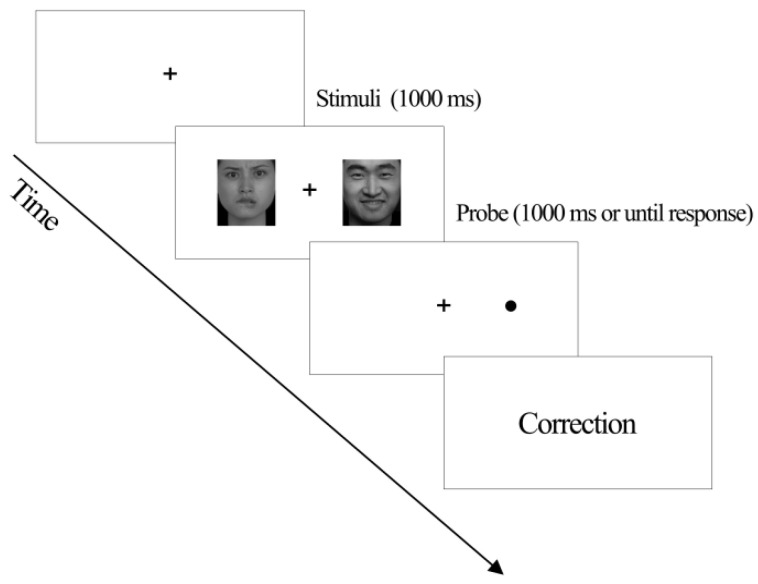
Stimulating steps in the task of emotional attentional biases.

Before the formal experiment, practice sessions with an unlimited number of practices were arranged. Participants could practice based on their conditions. After practice, participants started their formal experiments, with the task flow shown in Figure 3. An attentional bias score towards negative emotions was calculated with the sum of scores of responses under neutral-neutral face pictures subtracted from the sum of scores of responses under neutral-negative face pictures under the condition of inconsistency, with a higher attentional bias score towards negative emotions indicating more attention of the participant on negative emotional stimuli [27]. An attentional bias score towards positive emotions was calculated with the sum of scores of responses under neutral-neutral face pictures subtracted from the sum of scores of responses under neutral-positive face pictures under the condition of inconsistency, with a higher attentional bias score towards positive emotions indicating greater attention of the participant on positive facial expressions and a higher tendency of the participant to notice positive emotional stimuli [28].

### 2.4. Interventions

The actigaming used in this study is *JUST DANCE 2023* (developed by Ubisoft in 2022, Saint-Mandé, France) and runs on the Switch equipment (Nintendo, Kyoto, Japan). This game requires participants to follow the dance movements displayed on the electronic screen to achieve the effects of interaction and feedback. Also, an aerobic bicycle (MONARK 894E, made in Varberg, Sweden) was used in this study as a traditional exercise device. With different settings of resistance, this device requires participants to ride within a certain period to reach 60% of their maximum heart rates [29]. With mood changes caused by social interaction taken into consideration, irrelevant conversations among participants were minimized during all periods of intervention [30]. The total exercise time was 40 min, including 5 min of warm-up, 30 min of the main exercise, and 5 min of cool-down.

**Actigaming Intervention:**

To immerse participants in the actigaming dance *JUST DANCE 2023*, researchers provided a brief introduction and instructions on the operational methods of the game. Participants were required to follow the dance movements displayed on the electronic screen to facilitate interaction and feedback. Each participant completed a 30 min dance exercise. No specific gameplay instructions were given to encourage participants to engage in physical activity naturally, minimizing potential bias from the researchers.

The specific intervention plan is as follows:

**Exercise Type:** Participants will engage in a 30 min dance exercise using “*JUST DANCE 2023*.”

**Introduction:** Researchers introduced the game and its operational methods without detailed playing instructions.

**Objective:** The goal was to allow participants to follow their instincts while dancing, promoting a more authentic exercise experience.

**Duration of Exercise:** Each participant will complete a 30 min session of dance activity.

**Real-time Interaction:** Participants will receive visual feedback from the electronic screen based on their performance.

**Operation Procedure:** Each participant first selects a song with an appropriate difficulty level. During gameplay, the participant must hold a single Joy-Con controller in one hand. The system utilizes the accelerometer and gyroscope embedded in the controller to capture the amplitude, rhythm, and trajectory of hand movements, and subsequently applies computational algorithms to estimate the overall accuracy of the dance performance. Upon completion of each movement, the system provides immediate on-screen feedback in the form of performance ratings (e.g., OK, Good, Great, Perfect), which are accompanied by visual effects such as flashes, enhanced color displays, and animated icons. This real-time visual feedback not only facilitates the maintenance of rhythmic synchronization but also contributes to enhanced player engagement and motivational reinforcement.

**Song list for *JUST DANCE 2023*:** “About Damn Time” by Lizzo; “Running Up That Hill” by Kate Bush; “I Ain’t Worried” by OneRepublic; “Dance Monkey” by Tones and I; “Bang Bang” by Jessie J, Ariana Grande, and Nicki Minaj; “Mi Gente” by J Balvin and Willy William; “Savage Love (Laxed–Siren Beat)” by Jawsh 685 and Jason Derulo; “Levitating” by Dua Lipa featuring DaBaby; “Ghost” by Justin Bieber; and “Stay” by The Kid LAROI and Justin Bieber.

This intervention plan aims to enhance the enjoyment and effectiveness of the exercise by allowing participants to move freely and intuitively, fostering a more immersive and engaging experience in actigaming.

**Aerobic intervention:** To ensure participants maintained moderate intensity during a 30 min aerobic cycling exercise on a cycle ergometer (MONARK 894E, made in Sweden), laboratory staff adjusted the resistance of the bicycles based on real-time heart rate monitoring. Participants’ heart rates were monitored using portable heart rate monitors to ensure their exercise intensity was appropriate.

The specific intervention plan is as follows:

**Exercise Type:** Participants will engage in 30 min of aerobic cycling exercise.

**Initial Resistance:** It began with a 5 min warm-up at a resistance of 0.5 kp. Then, the resistance was adjusted to 1 kp, and participants were expected to maintain a rating of perceived exertion (RPE) in the range of 13–15. Finally, the resistance was returned to 0.5 kp for a 5 min cool-down.

**Real-time Monitoring:** Throughout the exercise, staff will continuously monitor participants’ heart rates to adjust the bicycle resistance as needed.

**Target Heart Rate Zone:** A target heart rate zone will be established based on participants’ maximum heart rates to ensure that exercise intensity remains at a moderate level (typically 50–70% of their maximum heart rate). Participants’ HR data were collected via Polar heart rate monitors (RCX3, made in Sweden).

**Duration of Exercise:** The entire duration of the exercise session is 30 min, divided into warm-up, main exercise, and cool-down phases.

**Data Recording:** Each participant’s heart rate data and exercise performance will be recorded for future analysis.

This intervention plan aims to optimize exercise outcomes and enhance participants’ comfort and motivation by alleviating their burden during the exercise process.

### 2.5. Exercise Load Control

For acute exercises with moderate intensity, a duration period of 20–40 min is necessary for the improvement of cognition and mood [10,31]. According to the guidelines of the American College of Sports Medicine, moderate intensity of an exercise should be set at the intensity under which heart rates of participants reach 60–69% of their maximum heart rates (HRmax), which are calculated as follows: HR max = 220-age (in years). To maintain their exercise intensity at a moderate level, all participants were monitored via Polar heart rate monitors (RCX3, made in Sweden). And were instructed to provide updates on their Rating of Perceived Exertion (RPE) levels every five minutes. For participants with heart rate or RPE levels exceeding the moderate intensity range, we adopted a reduction in the difficulty setting of the dance activity gaming, or the resistance of the ergometer bicycles was moderately reduced to adjust the participant’s exercise intensity.

### 2.6. Data Analysis

SPSS for Windows, Version 22.0 (SPSS Inc., Chicago, IL, USA) was used in this study to perform the statistical analysis. Continuous data were presented as mean ± standard deviation. Data distribution was assessed with the Shapiro–Wilk test for normality, while Levene’s test was used to check for homogeneity of variances. An independent-samples t-test was performed to compare the differences in maximum heart rates and average heart rates between these two interventions. Repeated-measures analysis of variance (ANOVA) was conducted to test the differences in scores of the POMS, ERQ, and emotional attentional biases task between these two interventions. A simple effect analysis was conducted to compare the differences between interventions when the interaction effect (interaction-effect: time (pre/post) ×  intervention (aerobic bicycle intervention/actigaming intervention) or main effect (time main effect or intervention main effect) was significant. A Greenhouse-Geisser correction method was used, and a Bonferroni correction was performed on post hoc pairwise comparisons for multiple comparisons, with a *p*-value less than 0.05 considered to be significant, while less than 0.01 was highly significant.

## 3. Results

### 3.1. Demographic Characteristics

Table 1 depicts sample characteristics. A total of 18 college students completed all testing. They were randomly divided into two groups (Group A and Group B). Briefly, the research sample consisted of 11 female and 7 male participants, with an average age of 24.17 ± 2.24 years, the majority of whom had body mass indices (BMIs) falling within the normal range.

**Table 1:** Demographic characteristics (n = 18).

	Group A	Group B	Total
Gender, female, n (%)	5 (55.6%)	6 (66.7%)	11 (61.1%)
Age (years), mean (SD)	24.33 (3.00)	24.00 (1.50)	24.17 (2.31)
Height (cm), mean (SD)	169.67(5.94)	168.78 (7.58)	169.22 (6.62)
Weight (kg), mean (SD)	64.21 (10.85)	58.17 (7.01)	61.19 (9.39)
BMI (kg/m^2^), mean (SD)	22.24(3.12)	20.37(1.40)	21.30 (2.53)

BMI: Body mass index.

### 3.2. Maximum Heart Rates and Average Heart Rates

Table 2 presents changes in the maximum heart rates and average heart rates of participants in the aerobic bicycle intervention and actigaming intervention. The study results show that the maximum heart rates and average heart rates of participants in the actigaming intervention are 159.11 ± 17.89 bpm and 131.08 ± 17.32 bpm, respectively, and the maximum heart rates and average heart rates of participants in the aerobic bicycle intervention are 136.69 ± 31.14 bpm and 114.31 ± 30.03 bpm, respectively, with the exercise intensities of all participants controlled within a moderate intensity range of exercise. It indicates that the differences in maximum heart rates and average heart rates of participants in the bicycle and actigaming intervention are not statistically significant. Normal distribution tests showed that the data for maximum heart rate and average heart rate corresponded to normal distributions.

### 3.3. Effects of Actigaming on Mood State and Emotion Regulation

**Figure 4:**
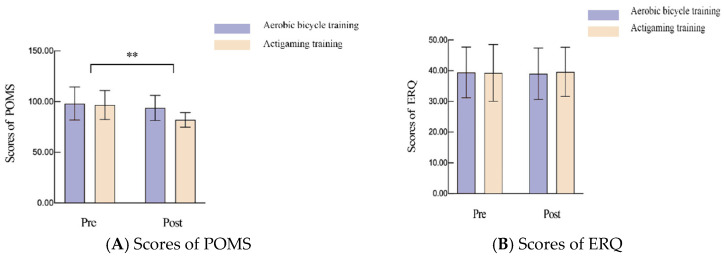
Effects of actigaming on scores of POMS and ERQ, * *p* < 0.05, ** *p* < 0.01.

Table 3 and Figure 4 present the results of a repeated measures ANOVA examining the effects of actigaming and exercise on participants’ mood state and emotion regulation. All groups demonstrated normal distribution and homogeneity of variance, satisfying the assumptions for ANOVA. The Profile of Mood States (POMS) scores exhibited a significant interaction effect (F(1,34) = 7.483, η^2^ = 0.180, *p* = 0.019), a non-significant main effect between interventions (F(1,34) = 0.654, η^2^ = 0.164, *p* = 0.433), and a significant main effect of time (F(1,34) = 10.122, η^2^ = 0.166, *p* = 0.001). Simple effect analysis indicated no significant baseline differences in POMS scores between the two interventions (*p* = 0.872, Figure 4A). However, post-test POMS scores were significantly lower among participants in the actigaming intervention compared to those in the aerobic bicycle intervention (*p* = 0.002). Furthermore, analysis of the Emotion Regulation Questionnaire (ERQ) scores revealed no significant interaction effect between interventions, nor significant main effects of intervention or time, indicating no significant differences in ERQ scores before and after either intervention.

### 3.4. Effects of Actigaming on Emotional Attentional Biases

**Figure 5:**
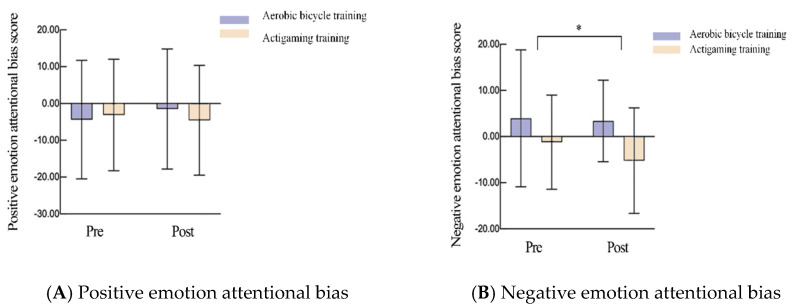
Effects of actigaming on the emotional attentional biases, * *p* < 0.05, ** *p* < 0.01.

Table 4 and Figure 5 present the results of a repeated measures ANOVA examining the effects of actigaming and exercise on emotional attentional biases. All data were normally distributed (*p* > 0.05) and met the homogeneity of variance assumption (*p* > 0.05), satisfying the assumptions for ANOVA. The results show that scores of attentional bias towards negative mood state exhibited a significant interaction effect between interventions (F (1, 34) = 4.223, η^2^ = 0.164, *p* = 0.027), a nonsignificant main effect of intervention (F (1, 34) = 6.762, η^2^= 0.166, *p* = 0.013) and a significant main effect of time (F (1, 34) = 0.657, η^2^ = 0.012, *p* = 0.415). Simple effect analysis indicated no significant baseline differences between the two interventions (*p* = 0.352, Figure 5A). However, post-test attentional bias scores towards negative emotions among participants in the actigaming intervention were significantly lower than those in the aerobic bicycle intervention (*p* = 0.001; Figure 5B). Furthermore, analysis of the Positive-emotion attentional bias score revealed no significant interaction effect between interventions, nor significant main effects of intervention or time, indicating no significant differences in Positive-emotion attentional bias scores before and after either intervention.

## 4. Discussion

In this study, a randomized crossover experiment was performed to investigate and compare the mood state benefits of actigaming exercise and traditional exercise. The study’s main results show that, compared with participants in the aerobic bicycle intervention, college students in the actigaming intervention may more quickly suppress cues related to negative emotions. We also found that participants in the actigaming intervention exhibited significantly enhanced positive mood state and reduced negative mood state after the intervention. However, we used a small data set and a single session; further research is needed with a larger sample size to confirm results.

Emotional attentional biases can influence how individuals perceive and interpret information, thus directly affecting their emotional experience [32]. Individuals with attentional bias shifting towards negative emotions will be more sensitive in their perception and treatment of negative information, thus making them prone to experiencing such negative emotions as anxiety, fear, or loneliness [33]. That is, if an individual is more likely to pay attention to negative information, he will feel depressed even in a positive environment [34]. On the contrary, individuals who tend to pay more attention to positive information will present stronger self-regulation and stress-tolerance abilities even in a predicament, which greatly affects their abilities to regulate and cope with emotions, thus influencing their decision-making behaviors [35]. Therefore, it is of particular importance for individuals to regulate their emotional attentional biases properly. This study shows that emotional attentional biases among college students can be positively regulated by both traditional exercise and actigaming, with actigaming showing a stronger regulatory effect than traditional exercise. Therefore, this study provides further evidence of the positive effects of exercise on attentional bias.

The attentional bias task performed in this study shows that college students can escape from negative emotion-related cues more quickly after their actigaming. Given the mutual influence between emotion and emotional attentional biases, both aspects can be regulated with exercise at the same time. This could be related to a large number of positive elements, like pictures and music in videos watched by college students during their actigaming. Scholars in the field of environmental psychology argue that compared with poor environments, rich environments are conducive to the improvement of cognitive functions of the brain. For instance, Lazarov et al. [36] proposed that music can facilitate the diversion of human attention from negative stimuli. Hutchinson et al. [37,38] argued that the use of music in physical exercises can induce positive emotions among exercisers or athletes and distract their attention from unpleasant feelings associated with physical exertion and fatigue. Exactly, actigaming can provide individuals with rich environmental elements. These rich environmental stimuli can stimulate the brain’s emotion regulation neural network, including such brain regions as the prefrontal cortex, amygdala, striatum, and anterior cingulate cortex [39,40,41,42,43], thus being beneficial for individuals to transform environmental negative stimuli into positive stimuli more quickly.

Compared to traditional exercise, actigaming demonstrates significant value in enhancing emotional well-being. On the one hand, research indicates that actigaming, particularly those related to dance, encourages users to engage more deeply in their workouts [44]. Participants are more actively involved in actigaming, as it stimulates their interest and motivation. In traditional exercise, monotony can lead to increased feelings of fatigue, thereby limiting the emotional benefits of the activity. In contrast, actigaming creates a joyful environment through gamified design and rich interactive experiences [45]. Studies have shown that this interactivity and enjoyment can promote the release of dopamine and endorphins in the brain, enhancing users’ feelings of happiness and satisfaction [46,47]. Moreover, actigaming provides immediate feedback and a sense of achievement, allowing participants to experience more enjoyment during their workouts rather than just fatigue. This positive emotional experience makes them more willing to continue participating, ultimately fostering an active lifestyle that further promotes overall mental health [45]. Actigaming is more than just a form of exercise; it serves as an effective tool for emotional regulation and offers new opportunities for mental health interventions. Our research indicates that cognitive changes associated with actigaming are not solely attributable to its interactive elements; they are also significantly influenced by participants’ effort. This relationship between actigaming and emotional well-being is multifaceted, encompassing both gamified interactions and individual commitment to physical exertion [20,45,48]. Thus, actigaming can play a vital role in enhancing emotional health while promoting active lifestyles.

Furthermore, our results show that attentional bias towards positive emotions shows no significant interaction with time across interventions, with no significant primary between-intervention or time effects. It indicates that actigaming did not significantly improve the tendency towards positive attention. This may be because the dance actigaming design—centered on rhythmic movement and immediate performance feedback—provides an incidental distraction from negative stimuli but does not include structured, positively valenced content (e.g., narrative rewards or social reinforcement) to actively draw attention toward positive cues [49]. Second, in healthy individuals, a strong baseline negative bias offers ample “downregulation” potential, whereas their positive bias is typically low or already near ceiling, leaving little room for a measurable single-session increase [50]. Due to the lack of comprehensive measurement of cognitive factors, the current findings should be interpreted with caution. Future research could employ neuroimaging techniques such as electroencephalography (EEG) or functional magnetic resonance imaging (fMRI) to further elucidate the underlying mechanisms. The findings of our study indicate that engaging in actigaming or bicycle exercise for a duration of thirty minutes can lead to enhanced positive affect and decreased negative affect among college students. This is consistent with the results of similar studies conducted among other populations [12,13,27,28,29,30,31]. Of course, it is undeniable that dance actigaming are another form of exercise. It has been widely accepted that exercise can stimulate the activity of the endorphin system, thus promoting the secretion of such neurotransmitters as dopamine [29] and endorphins, and the regulation of the secretion of these intrinsic hormones plays an important role in relieving negative emotions like stress [30]. In addition, according to the Self-determination Theory [31], when performing the actigaming, individuals could experience pleasure caused by the novel, diversified, and stimulating form of exercise. The reason is that the game’s rich graphics, sound, and music can stimulate individuals’ sensory abilities, and its novel interactive features can thereby promote their intrinsic motivation. For example, Jones et al. [32] verified that positive emotions of people stimulated by music and video, regardless of intensity, can be well promoted during exercise and after exercise. Furthermore, in their studies, Barnes et al. [33] found that video games can also provide adaptive and personalized virtual environments for anxious individuals, thus affecting their reward mechanisms and helping them reduce their anxious behaviors. Therefore, participants in actigaming can have a unique experience from which they can derive more enjoyment. Although a limited number of participants were included in this study, the positive effects revealed in this study can provide preliminary evidence for the investigation of actigaming improving the emotions of participants.

### 4.1. Implications for Research

This study highlights the potential of actigaming in regulating emotional attention bias and mood, serving as a foundation for future research. To build on these findings, subsequent studies should aim for larger sample sizes, ideally involving a more diverse participant pool, including various age groups, backgrounds, and fitness levels, to enhance the reliability and generalizability of results. (1) Future research should explore different formats of actigaming—such as virtual reality, augmented reality, or social multiplayer games—to determine which modalities are most effective for improving emotional states [51]. (2) The design of reward and feedback mechanisms based on actigaming is crucial. These mechanisms not only enhance participant motivation but also effectively promote the sustainability of exercise behaviors and emotional improvement. Reward mechanisms can include virtual rewards, point systems, or social recognition, while feedback mechanisms should provide real-time performance feedback and progress tracking to help participants clarify their goals and experience a sense of achievement. Further in-depth research into these characteristics will provide actigaming developers with insights for optimizing these mechanisms, ultimately helping to foster long-term user engagement and enhance overall emotional well-being. (3) To gain a clearer understanding of the value of actigaming in improving emotional states, large-scale interview studies are still necessary [52]. Interviews can delve into participants’ subjective emotional experiences, behavioral motivations, and needs, providing a solid empirical basis for optimizing actigaming design and targeted intervention strategies [53]. (4) Physiological mechanisms underpinning emotional responses—such as the release of dopamine and endorphins merit deeper investigation to paint a more comprehensive picture of actigaming’s emotional benefits. By illuminating these pathways, future studies can formulate precise guidelines for harnessing active gaming to enhance emotional well-being, thereby laying the groundwork for targeted, evidence-based therapeutic interventions.

### 4.2. Limitations

It should be noted that this research has the following limitations: (1) The small sample size of this study may limit the generalizability and extrapolation of the results. A small sample may lead to insufficient statistical power, making it difficult to detect true effects or differences. Additionally, individual variations can have a greater impact in a smaller sample, introducing random errors and reducing the reliability of the findings. Larger sample sizes should be applied in future studies to better investigate and compare the effects of actigaming and traditional exercises on emotional attentional biases. (2) Acute exercise interventions were performed in this study, presenting a short-term effect. Emotional improvements resulting from these acute exercise interventions are temporary, and their long-term effects cannot be fully determined. Meanwhile, it is also difficult to determine whether changes in emotions are caused by the influences of attentional bias on emotions or the influences of emotions on attentional bias. Therefore, subsequent studies should extend the intervention period to three months or longer and include extended follow-up assessments to rigorously evaluate the durability of emotional benefits. Conducting longitudinal research could help identify sustained effects on mood and emotional attention bias over time, providing insights into how regularly engaging with actigaming influences emotional health in the long term. (3) Due to constraints in experimental equipment, heart rate was not measured using strictly standardized tools (such as Polar heart rate monitors). The sensitivity and accuracy of the devices used may have interfered with heart rate readings, leading to potential data bias and affecting the validity of the results. Future research should further clarify the specific effects of actigaming on heart rate indicators. (4) We did not compute standardized effect sizes here because, in a small and heterogeneous pilot sample, such estimates can have unacceptably low precision. This will be addressed in a future, larger-scale validation study. (5) This study mainly relies on subjective or superficial behavioral indicators such as scales (e.g., POMS, ERQ) and behavioral tasks (emotional attention bias). To explore the potential mechanisms in more detail, supplementary methods such as EEG or fMRI can be considered to more precisely depict the differences in emotional processing between actigaming and traditional aerobic exercise from different temporal and spatial resolution levels. Finally, this study employed an assessor-blinded design; however, participants were not blinded, which may introduce bias into the results, especially for assessments using self-reported measures (e.g., POMS, ERQ). Future research could adopt ecological momentary assessment methods, such as experience sampling, after long-term interventions to enhance the reliability and ecological validity of the findings. Future research should include rigorously designed randomized controlled trials to confirm the effects of actigaming on emotional attention bias and mood, and should also implement long-term interventions to assess its lasting impact.

## 5. Conclusions

Actigaming is associated with a more favorable post-exercise mood and significantly attenuates attentional bias toward negative stimuli compared with aerobic cycling. Notably, the restricted sample size undermines the reliability of the findings. Consequently, the conclusions drawn from this study must be corroborated by additional empirical data to enhance their validity.

## Figures and Tables

**Table 2:** Maximum heart rates and average heart rates of participants in two interventions.

	Aerobic Bicycle Intervention	Actigaming Intervention	*t*-Test	*p*
Maximum heart rate (bpm)	136.69 ± 31.14	159.11 ± 17.89	−0.943	0.352
Average heart rate (bpm)	114.31 ± 30.03	131.08 ± 17.32	0.474	0.643

bpm: Beats per minute.

**Table 3:** Analysis results of mood state and emotion regulation.

	POMS Score		ERQ Score	
	Pre	Post	Pre	Post
Aerobic bicycle invention	98.11 ± 16.27	96.67 ± 14.23	39.44 ± 8.25	39.28 ± 9.25
Actigaming invention	93.78 ± 12.41	82.00 ± 7.18	39.00 ± 8.35	39.61 ± 7.98
Intervention main effect	F (0.654), η^2^ (0.180), *p* (0.433)		F (0.127), η^2^ (0.004), *p* (0.723)	
Time main effect	F (10.122), η^2^ (0.229), *p* (0.01)		F (3.283), η^2^ (0.088), *p* (0.08)	
Intervention × Time interaction-effect	F (7.483), η^2^ (0.193), *p* (0.019)		F (1.712), η^2^ (0.048), *p* (0.211)	

POMS: Profile of Mood States Questionnaire, ERQ: Emotion Regulation Questionnaire.

**Table 4:** Analysis results of emotional attentional biases.

	Negative Emotion Attentional Bias Score		Positive Emotion Attentional Bias Score	
	Pre	Post	Pre	Post
Aerobic bicycle invention	3.94 ± 14.84	−1.22 ± 10.20	−4.39 ± 16.11	−3.12 ± 15.16
Actigaming invention	3.38 ± 8.82	−5.22 ± 11.44	−1.50 ± 16.32	−4.56 ± 14.91
Intervention main effect	F (0.657), η^2^ (0.012), *p* (0.415)		F (0.758), η^2^ (0.008), *p* (0.421)	
Time main effect	F (6.762), η^2^ (0.166), *p* (0.013)		F (1.433), η^2^ (0.175), *p* (0.232)	
Intervention × Time interaction effect	F (4.223), η^2^ (0.164), *p* (0.027)		F (0.272), η^2^ (0.145), *p* (0.659)	

## Data Availability

For privacy and ethical reasons, data are available upon request from the corresponding author. To protect the confidentiality of potential and enrolled participants, personal information will be collected securely through online forms and interviews. All data will be managed by a designated researcher and stored on encrypted servers, with access limited to authorized personnel.

## Abbreviations

POMS	The Chinese Version of the Profile of Mood States Questionnaire
ERQ	The Chinese Version of the Profile of Emotion Regulation Questionnaire
CFAPS	Chinese Facial Affective Picture System
RPE	Rating of Perceived Exertion
ANOVA	Repeated Measures Analysis of Variance
BMI	Body Mass Index
